# Trajectories of Depressive Symptoms in Older Adults: Analysis of the Korean Retirement and Income Study

**DOI:** 10.1093/geroni/igaf122.4219

**Published:** 2025-12-31

**Authors:** Jin-kyung Lee, JaeWon Hyun, Bomgyeol Kim, Hun Kang, Hanna Lee, Seongmi Choi, JiYeon Choi

**Affiliations:** Yonsei University College of Nursing, Yonsei University, Seoul, Republic of Korea; Yonsei University, Seoul, Republic of Korea; Yonsei University College of Nursing, Yonsei University, Seoul, Republic of Korea; Yonsei University, Seoul, Republic of Korea; Yonsei University, Seoul, Republic of Korea; Daegu Catholic University, Daegu, Republic of Korea; Yonsei University, Seoul, Republic of Korea

## Abstract

Depressive symptoms are prevalent among older adults. It poses mental health challenges and increases the risks of frailty, disability, and even early mortality. With longer life expectancy, understanding how these symptoms change over time in later life is increasingly important. This analysis aimed to explore characteristics of depressive symptom trajectories among community-dwelling older adults using national survey data, the Korean Retirement and Income Study (KReIS). We analyzed 15,103 data reported by 2,717 older adults (72.77±5.07 years old on average, female: 60.43%) over six waves of the KReIS (2011-2021). As analytic methods, we used a latent growth mixture model (GMM) and longitudinal multilevel models (MLM). The four-trajectory model is chosen because of its best model fit: Constantly low (71.3%), constantly high (1.8%), increasing (16.3%), and decreasing (10.7%) depressive symptoms. Longitudinal MLM results demonstrate various effects of relevant factors by trajectory group. For example, in the group of reducing depressive symptoms (n = 244), the number of family members living together (β=-.66, p<.05), health satisfaction (β=-2.66, p<.001), hobby satisfaction (β=-.81, p<.05), and relationship satisfaction (β=-1.67, p<.01) were negatively associated with depressive symptoms. On the other hand, in the group of increasing depressive symptoms (n = 411), health satisfaction (β=-2.34, p<.001) and hobby satisfaction (β=-1.15, p<.001) were negatively associated, whereas the amount of medical expenditure (β=.49, p<.01) was positively related to depression. These results suggest that understanding heterogeneity in longitudinal depressive symptom patterns in older adults can guide efficient interventions. Personal and environmental factors may differ in significance, but relationship satisfaction appears central across all trajectories.

